# Saccular cyst with atypical presentation

**Published:** 2016

**Authors:** A Zamfir-Chiru-Anton, DC Gheorghe

**Affiliations:** * Department of Otorhinolaryngology, “G. Alexandrescu” Children Emergency Hospital, Bucharest, Romania; ** Department of Otorhinolaryngology, “M.S. Curie” Children Clinical Emergency Hospital; “Carol Davila” University of Medicine and Pharmacy, Bucharest, Romania

**Keywords:** saccular cyst, cricothyroid membrane, paramedian thyrotomy

## Abstract

Respiratory obstruction and stridor in infants and children are not uncommon. A rare cause of these sometimes life-threatening symptoms is the congenital saccular cyst.

**Objectives:** We present the case of a 5-year-old girl with a cervical tumor, which appeared after a laryngeal endoscopic surgery of a saccular cyst with two relapses and a particular local evolution of its recurrence through the cricothyroid membrane.

**Material and method:** The patient data has been reviewed over the entire follow-up period and a thorough an analysis of her investigations and surgery was performed.

**Results:** The unusual evolution of this case was marked by an atypical exteriorization – not found in the published literature. The surgical approach was external, by paramedian thyrotomy, with no further long-term recurrence.

**Conclusions:** An accurate diagnosis of saccular cysts can be made with the help of medical history, by an endoscopic visualization of the lesion and by the CT-scan imaging of the cervical region. Sometimes, saccular cysts can extend beyond laryngeal limits, determining fluid-filled tumors in the cervical region.

## Introduction

Laryngeal cysts are rare, achieved, or congenital benign lesions, which can affect all age groups. Congenital cysts of the larynx are an unusual cause of airway obstruction in newborns [**1**,**2**]. Some theories suggest that congenital anomalies are associated with an abnormal embryonic development of the larynx or an abnormal migration of the fourth branchial arch cells, which may form a sequestrated cyst [**3**]. Acquired cysts may be due to inflammatory, traumatic, or neoplastic obstruction of the saccular orifice. Their importance comes from producing dysphonia and/ or progressive respiratory distress.

Some studies reported that 20% to 30% of the laryngeal cysts in infants and children, especially those of the saccular type, have been associated with a variety of other congenital or developmental abnormalities [**2**,**4**]. Among these, Down’s syndrome, cystic fibrosis, cerebral palsy, cardiac anomalies, prematurity, hydrocephalus, microcephalus, high arched palate, micrognathia, conductive hearing loss, laryngomalacia, and vocal cord paralysis, are included [**15**].

DeSanto, Devine and Weiland classified all cystic laryngeal lesions into saccular, ductal and thyroid cartilage foraminal cysts [**5**].

Recently, Forte proposed a new classification dividing laryngeal cysts into two types based on their extent and the contained tissue [**1**]. A cyst that is radiologically and clinically confined to the larynx can be safely and completely excised endoscopically and is classified as type I. Those with extralaryngeal extension are classified as type II, further divided into IIa (with endodermal cells only) and IIb (with endodermal and mesodermal elements).

This article presents the case of a patient with a saccular cyst, which occurred 2 years after a successful endoscopic removal with an atypical exteriorization in the cervical region. This kind of exteriorization could not be found in the published literature, so, its evolution was discussed, and its surgical approach was presented.

## Case Report 

A 5-year-old girl was admitted in the Otorhinolaryngology Department for dysphonia, moderate inspiratory dyspnea, and anterior cervical tumefaction. The patient’s signs started 3 months before her presentation, with a progressive enlargement of her tumor.

The following data was retrieved from her medical history: at the age of 3, the patient was reported with moderate inspiratory dyspnea, frequent bouts of dysphonia and recurrent acute croups. The mother confirmed the dysphonia and appreciated its progressive course over the previous 2 years. 

**Fig. 1:**
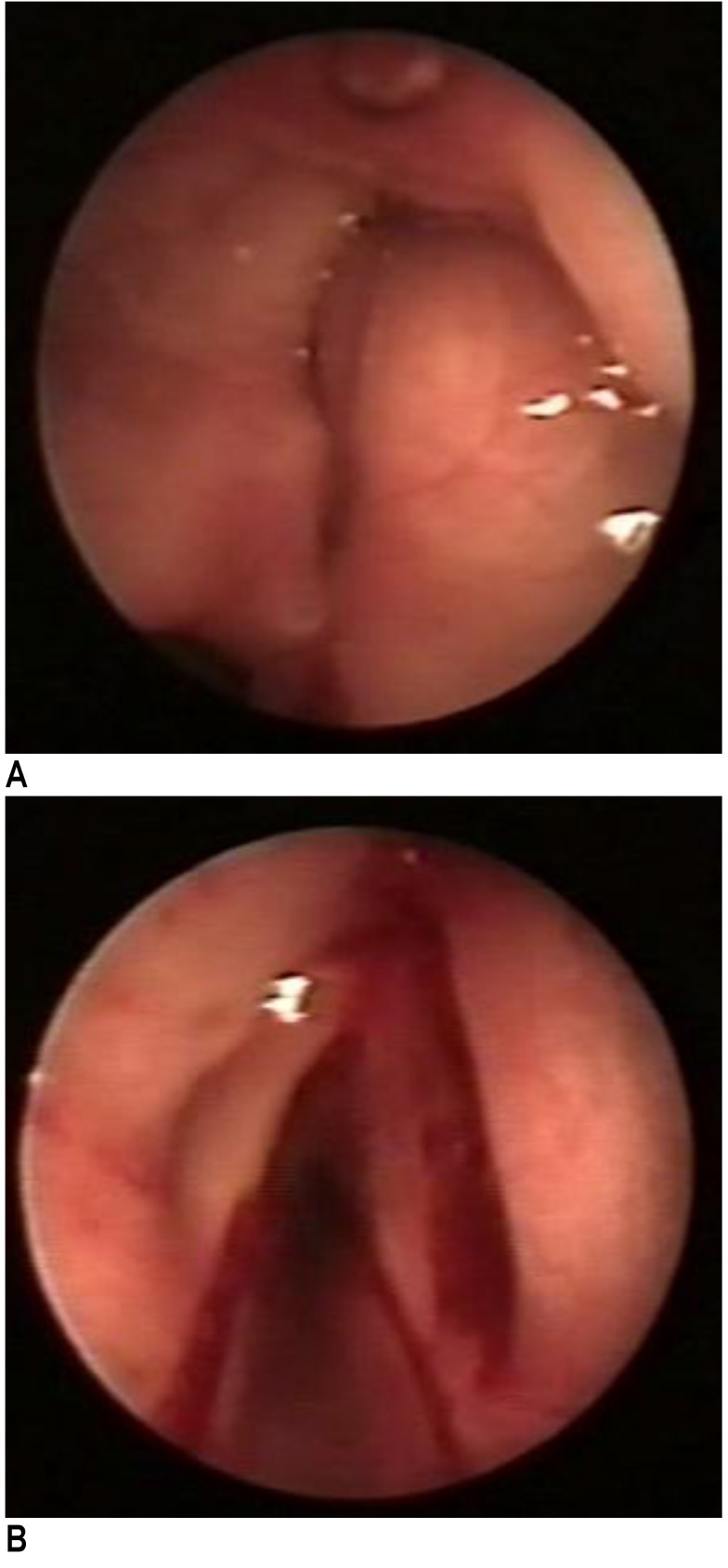
a. Cystic formation in the right ventricle, covered by normal mucosa; b. Intraoperative image after cyst excision

At the same time, the flexible laryngoscopy revealed the presence of a cystic tumor in the right ventricle covered by a normal laryngeal mucous membrane. A narrowing of the glottic space was recorded (**Fig. 1a**). 

A direct suspension laryngoscopy was conducted with a microscopic total removal of the right ventricular tumor. Upon its incision, a mucous, thick, yellowish fluid was noted, which was subsequently aspirated and the walls of the cyst were totally excised (**Fig. 1b**). The histologic examination reported the presence of a saccular cyst. The patient tolerated the procedure well and she was discharged 2 days after the surgery, with an alleviation of her dysphony.

**Fig. 2:**
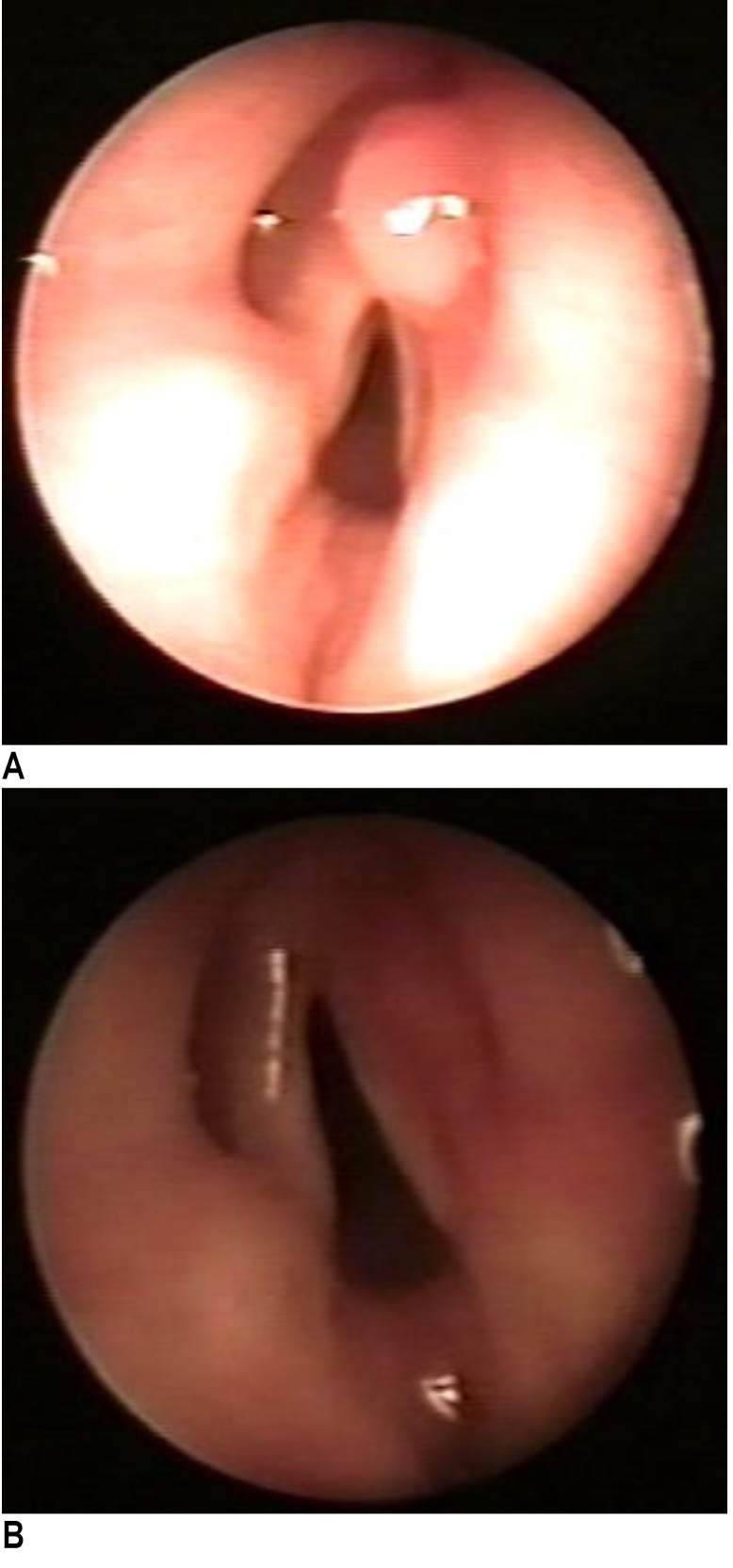
a. Cystic relapse 1 month after the first endoscopic surgery; b. Postoperative result after the second endoscopic excision

One month after the surgery, the patient returned for a check-up and a relapse of the cystic tumor was noted (**Fig. 2a**).

The patient was one again approached endoscopically and the tumor was completely removed. No recurrence was noted at the short follow-up (**Fig. 2b**). A slight dysphonia persisted.

**Fig. 3:**
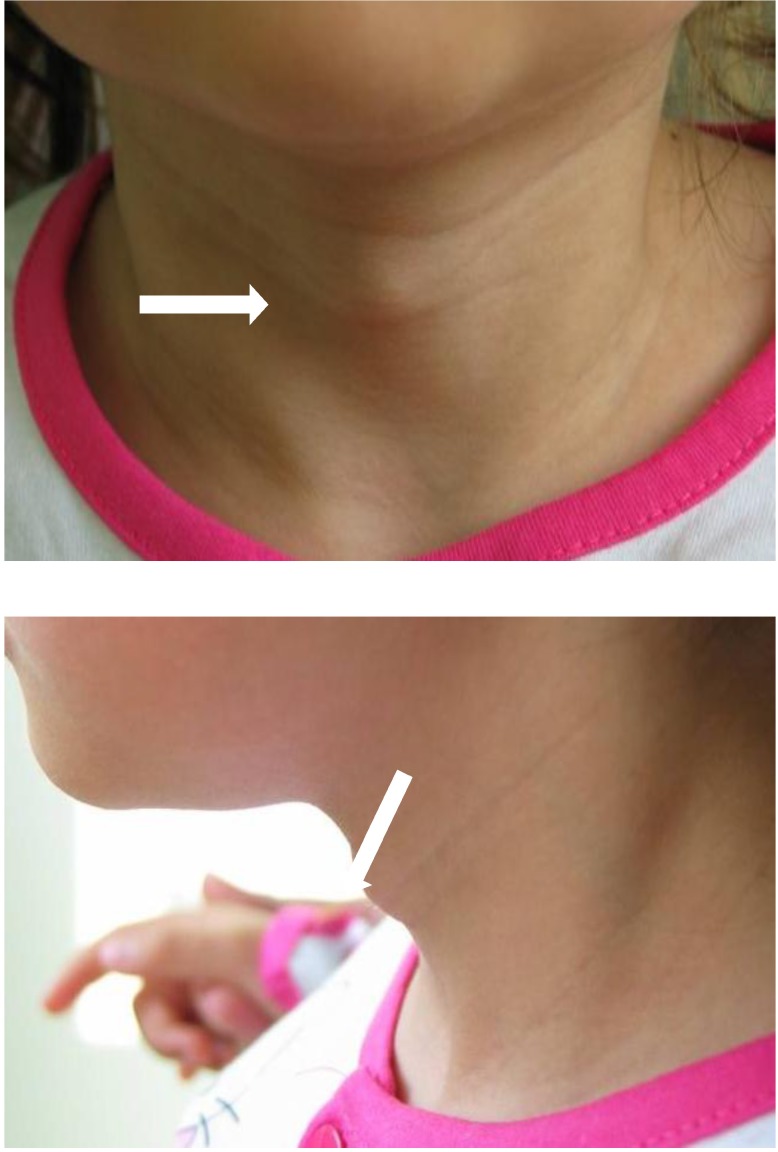
Cervical tumor appeared after 2 years after the endoscopic saccular cyst removal

At the current admission, the patient had an anterior cervical tumor located in the cricothyroid area, with normal covering skin (**Fig. 3**). Palpation revealed a soft, fluid-filled tumor of approximately 1/ 1cm, with a good mobility of the superjacent skin and firmly attached to the laryngotracheal structures. The patient had a minimal dysphonia. 

**Fig. 4:**
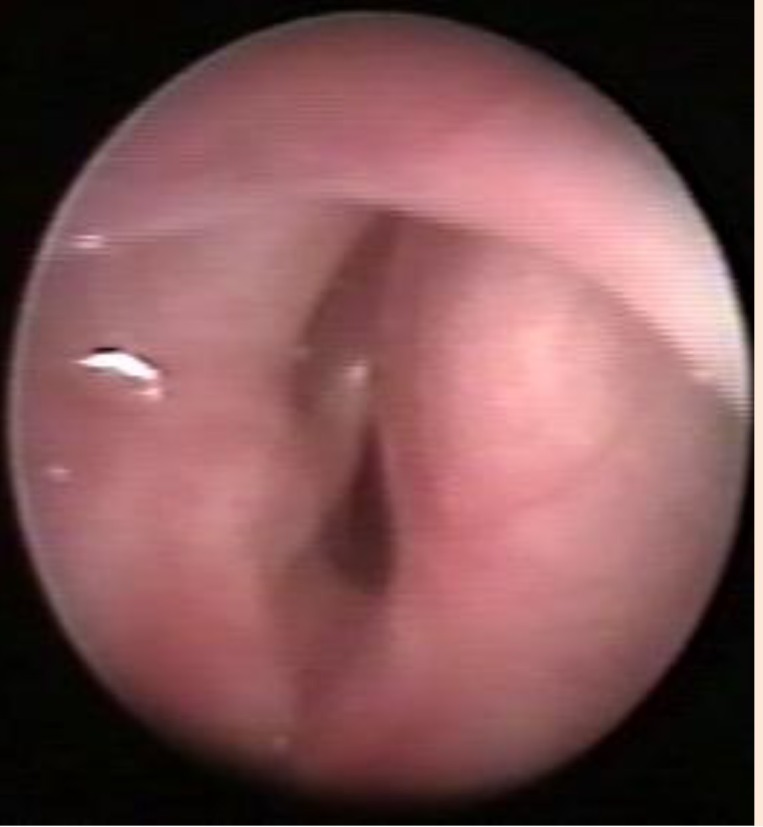
Endoscopic appearance 2 years after the first endoscopic saccular cyst removal

The flexible laryngoscopy showed a prominent right false vocal cord, with a cystic appearance of approximately 1.5 cm, partially covering the glottic space and the entire right vocal cord. The covering mucosa had a normal aspect (**Fig. 4**). The vocal cords were mobile.

**Fig. 5:**
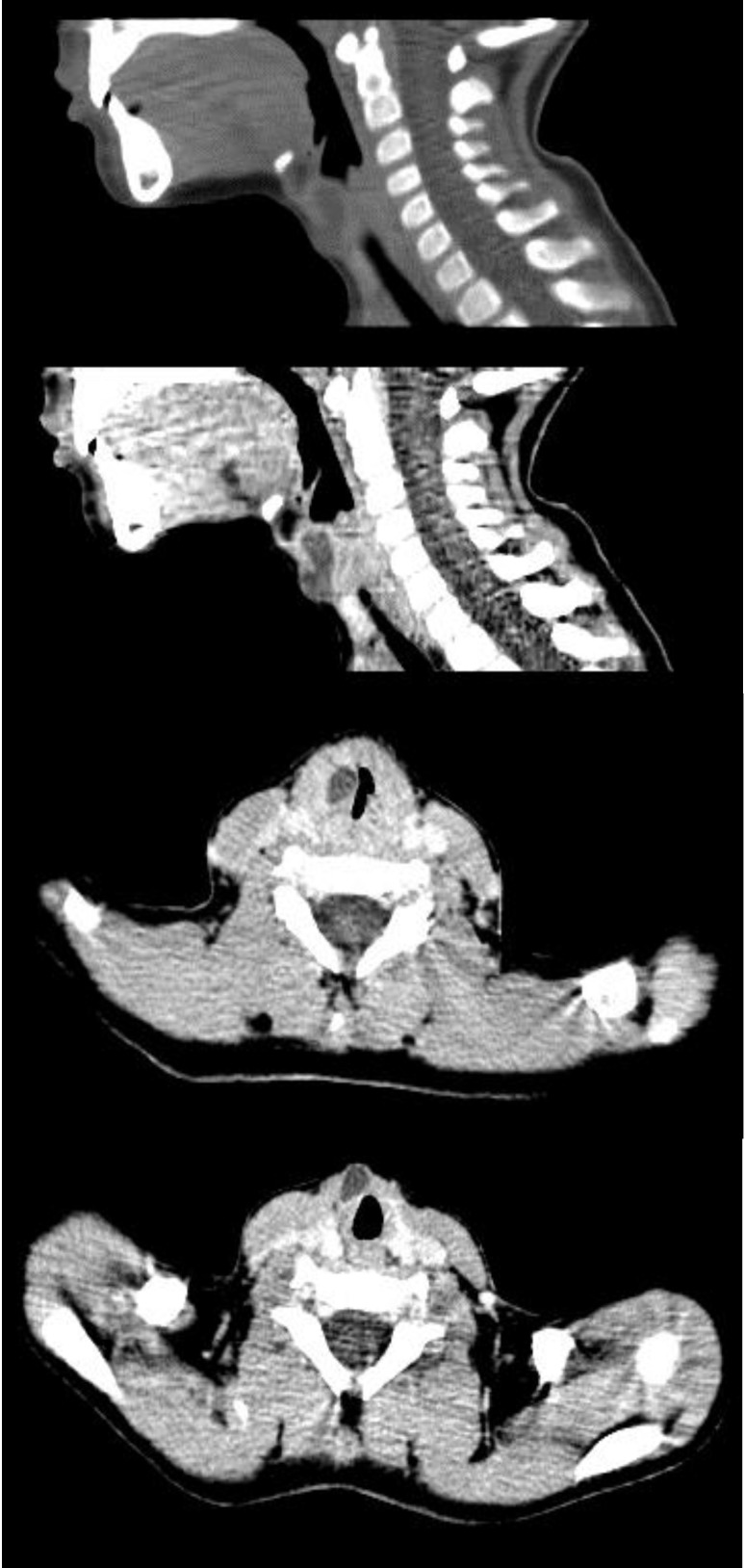
CT-scan of the patient, showing a laryngeal cyst extending through the cricothyroid membrane into the cervical region

The computer tomography of the anterior cervical area was performed, demonstrating a cystic tumor with fluid content, of approximately 3/ 1 cm, located in the middle and upper areas of the larynx, on the right side, which protruded through the cricothyroid membrane to the subcutaneous cervical region (**Fig. 5**). No cervical lymph nodes could be elicited.

The otorhinolaryngology clinical examinations and the imaging established the diagnosis of recurrent saccular cyst, with an atypical exteriorization (cricothyroid membrane).

**Fig. 6:**
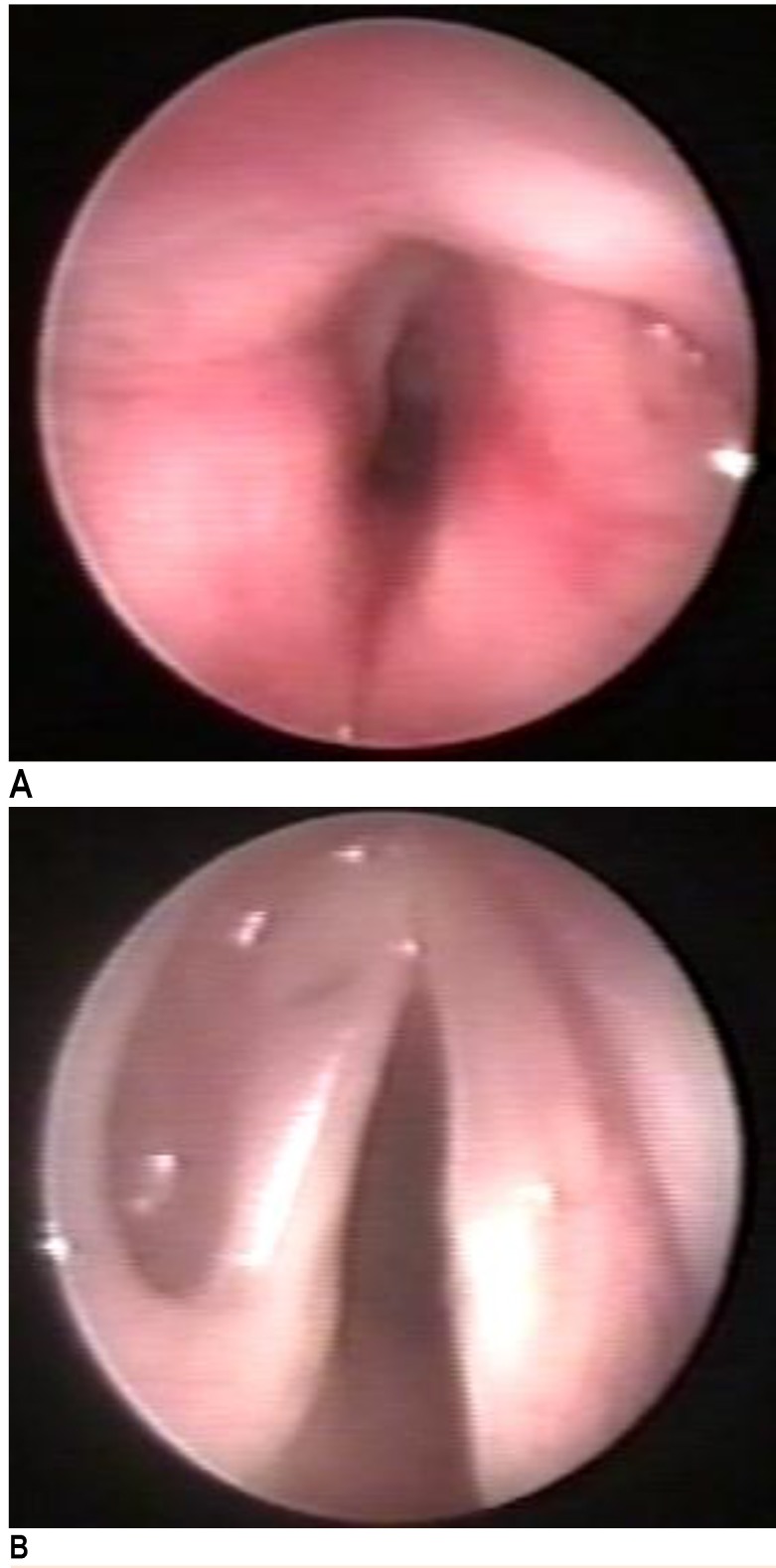
Postoperative image of the presented case: a. intraoperatively; b. 1 year after surgery

Surgery was decided based on those data. An external approach of the larynx was performed under general anesthesia with orotracheal intubation. Cervicotomy was started by a horizontal incision at the cricoid level. After retracting the musculocutaneous structures, the cystic formation was elicited; it partially dilacerated the cricothyroid membrane. The cyst was dissected upwards and inwards after the vertical incision of the thyroid cartilage, 6 mm on the right side (paramedian). The cystic wall was detached at a submucous level, in the right ventricle, without opening the endolaryngeal space. After the cyst removal, the endolarynx was examined by a flexible laryngoscopy (**Fig. 6a**). The thyroid cartilage was then sutured, the pre-laryngeal musculature was reconstructed, and the skin was closed. A 24 hours intubation was left in place. Decannulation was possible the next day under steroid treatment, with no difficulties. 

The paraffin-embedded preparations revealed a fibro-connective tissue, scarce striated muscular fibers, and glandular acini with sanguine suffusions; respiratory-type mucosa was also demonstrated at the cyst limits. A diagnosis of the laryngeal saccular cyst was established. 

The patient was given antibiotics and analgesics after surgery. The evolution was favorable, without complications. 

At the regular follow-up, the patient’s voice improved, but with a slight persisting dysphony. One year after the last surgery, the patient’s voice was almost normal and the flexible laryngoscopy showed a small scar lesion at the lateral edge of the right vocal cord and ventricle (**Fig. 6b**).

## Discussion

A discussion about laryngeal cysts requires a thorough understanding of the anatomy of the laryngeal ventricle and its appendage, the saccule. The ventricle is a deep fossa bounded by the false vocal fold and the true vocal fold. The anterior portion of the ventricle leads upward to a pouch known as the saccule [**2**]. The function of the saccule is believed to be the lubrication of the vocal folds with secretions from within its lumen. A saccular cyst occurs when the drainage pathway of the saccule becomes blocked or is congenitally not patent. The laryngeal saccular cyst probably represents 25% of all laryngeal cysts [**6**].

DeSanto also classified saccular cysts into anterior and lateral saccular cysts [**5**]. The anterior saccular cyst is located at the anterior ventricle, does not determine a significant obstruction, extends medially and posteriorly from the saccule, and protrudes into the laryngeal lumen between the true and false vocal cords. Lateral saccular cysts typically extend from false vocal cord to aryepiglottic fold posterosuperiorly and may bulge into the pharyngeal lumen and pyriform sinus [**5**]. Large lateral saccular cysts can extend into the lateral vallecula or bulge the medial wall of the piriform sinus. They can also herniate through the thyrohyoid membrane similar to a laryngocele and can appear in the neck region [**7**]. Inferior extension can take a cyst through the cricothyroid membrane into the paratracheal region [**1**]. 

Whereas the laryngocele is filled with air and is connected to the airway, a saccular cyst is filled with fluid. These cysts do not typically connect to the internal laryngeal lumen [**4**].

The age of the patient, the size of the cyst and the degree of obstruction of the laryngeal airway delineate the clinical presentation. In infants, the typical clinical signs of saccular cysts are generally noticed shortly after birth [**3**,**8**]. Stridor, muffled or weak cry and respiratory distress during feeding that results in regurgitation and cyanosis are typical findings. Laryngoceles may produce similar symptoms, but they are episodic [**4**].

The direct visualization of the pharynx, hypopharynx, larynx and subglottic area is crucial for the differential diagnosis in infants, who present with a stridor. Diagnosis is facilitated by CT-scan or magnetic resonance imaging, which will detect any extralaryngeal extension and help in planning the approach of the lesion [**9**]. Flexible transnasal endoscopy under local anesthesia may reveal other anatomic abnormalities and possible functional disturbances like vocal fold paralysis and laryngomalacia [**10**]. Direct laryngoscopy under general anesthesia is the second step for the detailed examination of the larynx and the subglottic region. 

Several different procedures have been used to treat saccular cysts in children. They range from the minimally invasive endoscopic needle aspiration [**11**] to more invasive procedures such as marsupialization [**10**], extended ventriculotomy, and the open laryngofissure approach [**4**]. The use of tracheotomy is usually discussed in relationship with the pediatric patient who presents with an airway obstruction. In cases of severe airway obstruction at birth, an immediate intervention is warranted with either tracheotomy or (if possible) intubation [**12**]. 

The repeated aspiration of the cyst with a needle is an atraumatic and successful method of treatment; however, subsequent literature has shown a high rate of recurrence with this method [**11**]. Currently, needle aspiration is considered a temporizing method in life-threatening airway obstruction [**9**]. 

The endoscopic treatment is the preferred method in the pediatric age group and is best suited for small endolaryngeal lesions. They allow a good precision and have a lower morbidity, thus they should be the initial choice for treating the most lateral saccular cysts of the larynx.

Kirse et al. recently described an endoscopic extended ventriculotomy procedure in which most of the lateral wall of the supraglottis, including the false vocal fold and the laryngeal ventricle, are resected [**13**]. With this approach, the surgeon opens the saccule at its natural drainage point. This procedure allows a continued decompression. 

Several authors have advocated the use of the CO2 laser. CO2 laser-assisted endoscopic treatment consists of the complete removal or extensive marsupialization of the cyst [**14**]. 

To prevent recurrences after repeated endoscopic surgeries, a complete removal of the cyst can be achieved through an external approach [**15**]. However, an external approach may be preferred as the first surgical step depending on the size and the location of the cyst [**9**]. The external approach may be performed through lateral cervical approach [**15**], laryngofissure or paramedian thyrotomy [**9**]. 

## Conclusions

Saccular cysts can sometimes present recurrences, even after an apparently complete excision. The atypical exteriorization into the cervical region, through the cricothyroid membrane, has not yet been communicated in the published literature.

The mechanism of cyst development is unknown, but can be linked to local mucous producing glands anatomy. 

The removal of the cyst can be done easily with a minimum morbidity for the patient, by endoscopy. The recurrence of our patients’ cyst 2 years after surgery showed an incomplete excision technique even if performed by using the operating microscope.

The CT-scan imaging, using contrast has an important value in confusing cases, especially in revealing the cyst location, extension, and structure.

The external approach of the saccular cysts by vertical paramedian thyrotomy and total removal of the cyst, can avoid the laryngeal mucosa opening and the need for a tracheotomy.

The excellent collaboration between several specialists (otorhinolaryngologist, radiologist, and anesthetist) could yield the best results when dealing with infant/ small children airway disease. 
